# CF Fungal Disease in the Age of CFTR Modulators

**DOI:** 10.1007/s11046-021-00541-5

**Published:** 2021-04-04

**Authors:** Amelia Bercusson, George Jarvis, Anand Shah

**Affiliations:** 1 Cystic Fibrosis Unit, University Hospital Southampton NHS Foundation Trust, Southampton, UK; 2 Respiratory Medicine, Royal Brompton and Harefield NHS Foundation Trust, London, UK; 3 Department of Infectious Disease Epidemiology, MRC Centre of Global Infectious Disease Analysis, School of Public Health, Imperial College London, London, UK

**Keywords:** Cystic fibrosis, *Aspergillus fumigatus*, Fungi, Antifungal

## Abstract

Fungi are increasingly recognised to have a significant role in the progression of lung disease in Cystic fibrosis with *Aspergillus fumigatus* the most common fungus isolated during respiratory sampling. The emergence of novel CFTR modulators has, however, significantly changed the outlook of disease progression in CF. In this review we discuss what impact novel CFTR modulators will have on fungal lung disease and its management in CF. We discuss how CFTR modulators affect antifungal innate immunity and consider the impact of Ivacaftor on fungal disease in individuals with gating mutations. We further review the increasing complication of drug–drug interactions with concurrent use of azole antifungal medication and highlight key unknowns that require addressing to fully understand the impact of CFTR modulators on fungal disease.

## Cystic Fibrosis Fungal Disease: An Introduction

Cystic fibrosis is an inherited multi-system disorder which manifests in the lungs as chronic airways infection and inflammation leading to bronchiectasis and, if left untreated, progressive respiratory failure and death. Although bacteria are the dominant airway pathogens in the majority of the CF population, fungi are increasingly being recognised as having a role in the pathophysiology of CF lung disease.

*Aspergillus fumigatus* is the most common and clinically significant fungus isolated from CF patients, but published prevalence rates for colonisation range widely from less than 5% to 60% [1]. This variability reflects differences in definitions of colonisation, patient demographics, treatment regimens, frequency and type of sampling as well as laboratory culture technique [2, 3]. Age, inhaled corticosteroids and antibiotic use (macrolide and inhaled antibiotics) have been identified as risk factors for *Aspergillus* colonisation. Although causality is unproven, it is hypothesised that earlier aggressive use of inhaled antibiotics as antimicrobial prophylaxis could potentially predispose to increased fungal colonisation rates [4, 4]

The clinical significance of *Aspergillus fumigatus* in CF depends on the host’s immunological response. Baxter et al. [6] proposed a classification for *Aspergillus* lung disease in CF based on microbiology (culture, sputum galactomannan, *Aspergillus* real-time polymerase chain reaction) and serology (*A. fumigatus*-specific IgE and IgG), which defined four clinical groups: allergic bronchopulmonary aspergillosis (ABPA), *Aspergillus* sensitisation, *Aspergillus* colonisation and *Aspergillus* bronchitis.

ABPA is a Th2- and IgE-mediated hypersensitivity response to *Aspergillus* with high prevalence (8.9%) in the CF population [7]. The US CF foundation and the International Society for Human and Animal Mycology (ISHAM) have published diagnostic criteria for ABPA in CF (Table 1) [8, 9]. In both criteria, diagnosis depends on elevated total and *Aspergillus*-specific IgE together with at least one (US CFF) or two (ISHAM) of the following: raised *Aspergillus*-specific IgG, consistent radiological changes or raised eosinophils (ISHAM criteria only). Evidence for the impact of ABPA on lung function in CF has been inconsistent. In an early retrospective study, Kraemer et al. [10] demonstrated a negative impact on several lung function parameters similar to the impact of chronic *Pseudomonas* infection. More recently, De Baets et al. [11] assessed *Pseudomonas*-negative CF patients in a retrospective case–control study and found that those with ABPA experienced a significant decline in lung function over 2 years leading up to diagnosis compared to control cases whose lung function remained stable. By contrast an ECFS registry study found only a modest ABPA-dependent difference in FEV1 at entry into the study that did not alter during the 3-year follow-up [12].

**Table 1:** Table summarising proposed criteria for diagnosis for allergic bronchopulmonary aspergillosis and *Aspergillus* bronchitis in Cystic Fibrosis

Classic case	Minimal diagnostic criteria
*Criteria for diagnosis of allergic bronchopulmonary aspergillosis in patients with cystic fibrosis*	
Acute or subacute clinical deterioration that is not attributable to another aetiology (cough, wheeze, exercise intolerance, exercise-induced asthma, decline in pulmonary function, increased sputum)	Acute or subacute clinical deterioration that is not attributable to another aetiology (cough, wheeze, exercise intolerance, exercise-induced asthma, change in pulmonary function or increased sputum production)
A serum total IgE level of > 1,000 IU per ml (2,400 ng/ml) unless patient is receiving systemic steroids (requires retest when steroid treatment is discontinued)	A serum total IgE level of > 500 IU per ml (> 1,200 ng/ml). If ABPA is suspected and the total IgE level is 200–500 IU per mL, repeat testing one to three months is recommended. If patient is taking steroids, repeat when steroid treatment is discontinued
Presence of IgE antibodies to *A. fumigatus* in vitro or immediate cutaneous hypersensitivity to aspergillus	Immediate cutaneous reactivity to *A. fumigatus* (prick test wheal > 3 mm with surrounding erythema, off systemic antihistamines) or in vitro demonstration of IgE antibody to *A. Fumigatus*
Precipitating antibodies to *A. fumigatus* or serum IgG antibody to *A. Fumigatus*	*One of the following*:
	Precipitating antibodies to *A. fumigatus* or serum IgG antibody to *A. fumigatus*
	New or recent infiltrates ( or mucus plugging) on chest radiology or computed tomography that do not respond to antibodies and standard physiotherapy
	New or recent abnormalities on chest radiography (infiltrates or mucus plugging) or computed tomography (bronchiectasis) that do not respond to antibiotics and standard physiotherapy
*Criteria for Aspergillus Bronchitis*	
Microbiology	Repeat sputum culture for *Aspergillus sp*
	Positive sputum galactomannan
Symptoms	Chronic (> 4 weeks) pulmonary symptoms (chronic productive cough, tenacious mucus production, dyspnoea and difficult airway clearance)
Absence of semi-invasive disease	Absence of significant tissue invasion and lung parenchymal destruction (e.g. cavity formation)
Serology (Bronchoscopy findings)	*Aspergillus* IgG antibody detectable in serum
	Negative IgE (lack of allergic response)
	Mucoid impaction, thick tenacious sputum with bronchial plugging, bronchial erythema (touch bleeding) and/or ulceration
	Superficial invasion of mucosa by *Aspergillus* hyphae

The proposed classification of *Aspergillus* bronchitis relates to microbiological colonisation with sputum galactomannan positivity and a positive *Aspergillus*-specific IgG but negative IgE response (Table 1). No formal diagnostic criteria have been published and the immunological host response in this subgroup, the prognostic implications and effect of antifungal therapy are as yet not well defined. Clinical presentation with deterioration despite ≥ 2 courses of antibiotics with exclusion of new bacterial growth and response to antifungal therapy has been proposed as confirmation of *Aspergillus* bronchitis [13].

The impact of non-ABPA manifestations of *Aspergillus*-related disease on lung function has been harder to determine with a wide variation in definitions used and a failure to distinguish between sub-groups. Studies that compared *Aspergillus*-sensitised to *Aspergillus* negative groups show a negative effect on lung function; however, most studies comparing colonised versus non-colonised populations have not identified *Aspergillus* as a risk factor for lung function decline [14–16].

As well as *Aspergillus*, individuals with CF are also susceptible to colonisation with other filamentous mould, in particular *Scedosporium* species and *Exophiala dermatitidis*, which can dominate the fungal airway community [17]. The susceptibility, implication and relevance of colonisation with these emerging pathogens are as yet unclear, with multicentre cross-sectional analyses recently not identifying a particular severity trait [18, 19]. Further longitudinal cohort studies are necessary to fully understand pathogenicity and implication on long-term outcome.

## Fungal Immunity in Cystic Fibrosis

Cystic fibrosis is caused by mutations in the cystic fibrosis Transmembrane Conductance Regulator (CFTR) gene. CFTR encodes a cyclic AMP-activated chloride channel highly expressed in epithelial cells but also localised to the surface and endosomal membranes of immune cells [20, 21]. The absence of functional CFTR has a profound impact on innate and adaptive immune responses to inhaled fungal pathogens and it is this dysregulated host response which plays a key role in determining the pathogenicity of fungal exposure in CF.

### Innate Immunity

The CFTR ion channel conducts Cl^−^ and HCO3^−^ ions across the cell membrane, with loss of function interfering with normal anion transport and, through its regulatory effect on the ENaC sodium channel, sodium reabsorption. This disruption across airway epithelial cell membranes results in abnormal hydration of airway surface liquid (ASL) and impaired mucociliary clearance, a key mechanism for preventing fungal colonisation [22]. Fungi retained within the airways are then liable to be sensed by epithelial cells and airway resident immune cells responsible for ingesting and clearing pathogens. In the CF airway, signalling pathways are triggered to recruit further inflammatory cells and activate adaptive immune responses. There is known to be variability in predisposition to fungal disease in CF, with genetic susceptibility and HLA-DRB1 alleles shown to be associated with ABPA [23].

CFTR-deficient epithelial cells show impaired phagocytosis and killing of *Aspergillus* conidia and increased rates of apoptosis in vitro and impaired fungal clearance in vivo [24]. IL-8 secretion, a key neutrophil chemoattractant, is increased as is NLRP3 inflammasome activation [25, 26]. Changes in TLR4 expression, cytokine production, phagosomal acidification, microbicidal activity, autophagy and inflammasome activation have all been demonstrated in CF macrophages [27–29]. Almost all of these findings, however, are based on work using bacterial stimulants, with little data on responses to fungal stimuli. A previous study has, however, shown impaired autophagy in lung macrophages isolated from *A. fumigatus* infected CFTR-deficient mice, alongside increased *Aspergillus*-dependent NLRP3 inflammasome activation and IL-1β release [25].

Neutrophils dominate the CF airway drawn by chronic microbial infection and augmented secretion of cytokines and chemokines including IL-1β, IL-8, TNF-α and LTB4 [30]. Once present, CF neutrophils show reduced rates of apoptosis, persisting in the airways [31]. Reactive oxygen species (ROS) release in response to *Aspergillus* infection is increased and has been linked to disease progression [32]. *Aspergillus* is also known to trigger formation of neutrophil extracellular traps (NETs), which are important for controlling hyphal growth [33]. Excess release of NETs have been identified as contributing to airway obstruction and diminished lung function in CF [34].

### Adaptive Immunity

T lymphocytes play a key role in fungal immunity. Th1 CD4 + T cells enable inflammation and fungal clearance, whereas Th2 cells drive allergic inflammation with T regulatory cells having an immunomodulatory role [35–37]. A tendency to develop a Th2 response to *Aspergillus* has been demonstrated in CF murine models and in the peripheral blood of CF patients with ABPA [38, 39]. Impaired IFN-γ and IL-10 release by T helper cells from CF patients suggests skewing away from a protective Th1 response [40]. A recent study has additionally shown the importance of *Aspergillus-*specific Th17 cells in driving ABPA in CF with increased blood levels during exacerbations and a reduction following antifungal therapy [41]. *Aspergillus*-specific Th17 cells additionally recognised a restricted set of cross-reactive proteins including *Candida albicans* potentially driving pathogenicity although the full implications are as yet unclear.

## CFTR Modulators

Over the past few years, a number of small molecule therapies capable of enhancing the functional expression of specific CFTR mutations have been released onto the market and still more are under development. They are known collectively as CFTR modulators but can be classified into five main groups: potentiators, correctors, amplifiers, read-through agents and stabilisers. The potentiator Ivacaftor (Kalydeco®) was the first to be approved by the FDA in 2012. Its mechanism of action is to increase the “open probability” (*P*_o_) of the CFTR ion channel at the cell surface. As such, Ivacaftor was originally licenced only for Class III gating mutations, which represent ~ 5% of the CF population, with subsequent expansion to a number of residual function mutations [42]. Several phase 3 trials demonstrated safety and efficacy with improvement in lung function and reduction in exacerbations [43].

Lumacaftor/ivacaftor (Orkambi®) and tezacaftor/ivacaftor (Symdeko®) are both combination corrector–potentiator drugs. The lumacaftor and tezacaftor entities work in synergy with ivacaftor to correct protein misfolding prior to its transport to the cell surface, where its resultant activity is enhanced by ivacaftor’s effect on anion channel function. Combination therapy enables efficacy in improving lung function (6.8% relative improvement in FEV1), quality of life and reducing exacerbations in a much larger cohort of patients, namely Phe508del homozygotes [44, 45], as well as Phe508del/residual function heterozygotes [46].

Finally, the most recently licenced modulator is elexacaftor/tezacaftor/ivacaftor (Trikafta® or Kaftrio®). This is a combination of two correctors and ivacaftor and has been licenced for use in Phe508del homozygotes and the 30% of patients who have one copy of Phe508del and one copy of a “minimal function” mutation [47]. For the former group, triple therapy was compared to tezacaftor/ivacaftor in a phase 3 trial and showed a relative 10% increase in FEV1, reduction in sweat chloride and improvement in quality of life scores [48]. For the Phe508del heterozygotes, triple therapy was compared to placebo and demonstrated an average 14% increase in FEV1 and 63% reduction in pulmonary exacerbations as well as improvements in sweat chloride and quality of life scores [49].

## CFTR Modulator Impact on Fungal Colonisation and Disease

An important question in this new era of CFTR modulators is what impact these drugs will have on fungal lung disease and its management in CF. There is some early evidence from registry studies of a beneficial effect of Ivacaftor on *Aspergillus* colonisation rates [50–52]. Of note, these studies have identified a poly-microbial effect with significant reductions in *Pseudomonas aeruginosa* and *Staphylococcus aureus* prevalence rates as well, in part likely related to improved mucociliary clearance [53]. There is as yet no data on the impact of combination modulator therapies on microbial colonisation rates, though one might expect similar beneficial effects, with subsequent effects on sensitisation and ABPA.

Given the effects of CFTR dysfunction on the host immune cell response, it is likely that these modulators will also impact the dysregulated host response that drives fungal disease in CF, however the magnitude of response and clinical effect is as yet unknown. An early study by Pohl et al. showed that ivacaftor restores degranulation and improves bacterial killing by neutrophils [54]. Since then, others have demonstrated an ivacaftor-dependent normalisation of CF neutrophil phenotype with reduced expression of activation and adhesion markers and recovery of normal apoptosis rates [31, 55].

**Fig. 1:**
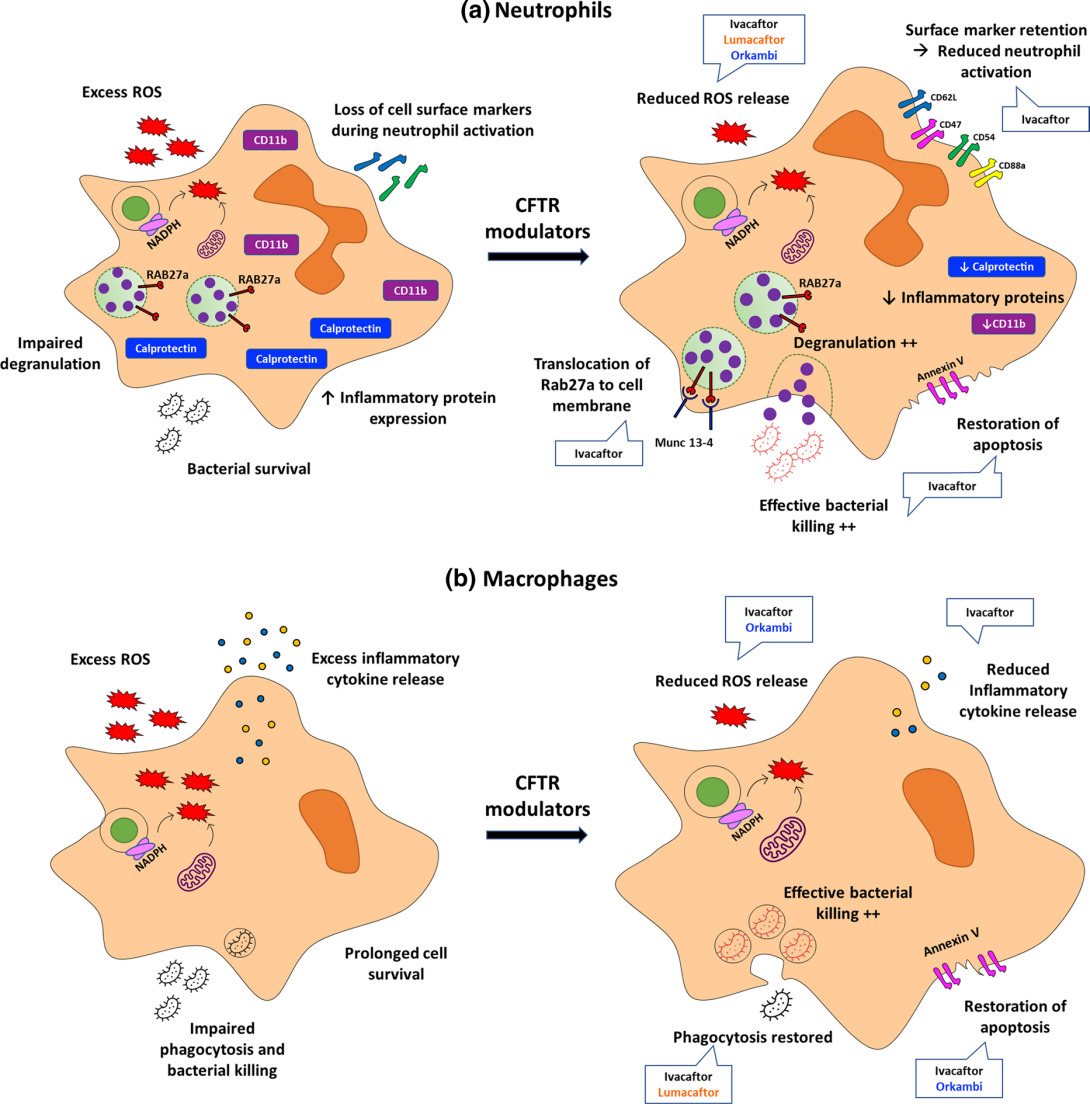
**a** Ivacaftor-treated neutrophils demonstrate: reduced reactive oxygen species (ROS) release; restored degranulation and bacterial killing; reduced expression of inflammatory proteins and retention of surface markers normally lost during neutrophil activation and normalised rates of apoptosis. Lumacaftor and Orkambi also reduce ROS release. **b** Ivacaftor-treated macrophages demonstrate: reduced ROS and inflammatory cytokine release; restored phagocytosis and bacterial killing and normalised rates of apoptosis. Lumacaftor reduces ROS release and restores phagocytosis and bacterial killing. Orkambi reduces ROS release and restores normal rates of apoptosis

The impact of CFTR modulators on macrophage function is more variable and modulator-type dependent. Barnaby et al. [56] showed that lumacaftor alone, but not ivacaftor alone, restored defective CF macrophage phagocytosis and *Pseudomonas* killing. When ivacaftor was added to lumacaftor in the form of Orkambi®, however, this cancelled lumacaftor’s beneficial effects. By contrast, both Ivacaftor alone and Orkambi® reduced macrophage secretion of pro-inflammatory cytokines, whereas lumacaftor alone did not. In another study, both ivacaftor and Orkambi® normalised increased macrophage apoptosis rates, but only ivacaftor normalised phagocytosis and *Pseudomonas* killing and reduced inflammatory cytokine release [57]. Only one study has examined the effect of CFTR modulators on immune responses to fungal infection. All three modulators studied, ivacaftor, lumacaftor and Orkambi® reduced the exaggerated ROS production seen in CF mononuclear cells and neutrophils infected with *Aspergillus* [58]*.* Figure 1 provides an illustration of the impact of CFTR modulators on antimicrobial immunity.

Any impact of CFTR modulators on antifungal immunity will, however, need to be balanced with susceptibility to pathogenic fungi as life expectancy increases, with resultant potential longer duration of inhaled antibiotic exposure in individuals with pre-existing structural lung disease, and additional effects of older age on the host immune response. Aspergillus colonisation rates have additionally been shown to be associated with non-tuberculous mycobacteria and bacteria such as *Pseudomonas aeruginosa*. As such, there may be ongoing susceptibility to fungi in CF individuals with pre-existing lung disease and *Pseudomonas aeruginosa* or NTM colonisation and requirement for inhaled antibiotic prophylaxis, but reduced susceptibility in individuals with minimal existing structural lung disease prior to CFTR modulator initiation. Further longitudinal prospective cohort studies will thus be needed to determine the long-term cumulative implications of CFTR modulator therapy on fungal colonisation, sensitisation and disease prevalence in CF.

## CFTR Modulator–Antifungal Interactions

A complication of CFTR modulator therapy is the potential for drug–drug interactions with the most commonly used group of antifungal drugs, the triazoles. Ivacaftor, tezacaftor and elexacaftor are all primarily metabolised via CYP3A-mediated oxidation, whereas Lumacaftor is not extensively metabolised and is mostly excreted unchanged in the faeces. Lumacaftor is, however, a strong CYP3A inducer so drug–drug interactions with Orkambi® can be more difficult to predict.

The triazoles are all CYP3A inhibitors and so CFTR modulator dose adjustments have to be made [59, 60]. Itraconazole, posaconazole and voriconazole are all considered strong inhibitors and so a dose reduction to a single morning combination tablet twice a week for Symdeko® and Trikafta/Kaftrio® and a single ivacaftor tablet twice weekly for Kalydeco®, is advised. There is to date, however, limited available data on the bioavailability of CFTR modulator therapy. The novel azole isavuconazole is considered to be only a moderate CYP3A inducer and so less severe dose reductions can be made [61]. Alternate-day single doses of the combination and ivacaftor tablets are advised for Symdeko® and Trikafta/Kaftrio® and once daily ivacaftor for Kalydeco®.

The management of these drug–drug interactions, however, is significantly complicated by the variability of triazole pharmacokinetics [62–64]. Gastrointestinal absorption of itraconazole and posaconazole is strongly influenced by drug formulation, gastric pH and co-administration with food and frequency of administration [65]. Voriconazole meanwhile exhibits nonlinear and saturable pharmacokinetics, making drug exposure after dose adjustments unpredictable. The area under the curve (AUC) varies according to age, sex and CYP2C19 polymorphism [66]. Current first-line treatment for CF-related fungal disease is inconsistent, but more commonly itraconazole or voriconazole [67]. Therapeutic drug monitoring has a key role in improving patient outcomes, preventing increasing prevalence of antifungal azole resistance and minimising toxicity, but studies have shown widespread prevalence of subtherapeutic azole levels in CF, potentially resulting in ineffective CFTR modulator therapeutic dosing [68–71]. To date, there is little real-world data on the implication of azole interaction on CFTR modulator dosing and efficacy. Isavuconazole, a novel azole with more predictable pharmacokinetics and reduced drug–drug interactions, may present a better therapeutic choice, but there is as yet limited data on CFTR modulator interaction.

Alternative antifungal therapy such as the echinocandin (e.g. Caspofungin) and polyene (e.g. amphotericin) classes do not interact with CFTR modulator therapy but currently are only available in intravenous form, making them impractical in a chronic setting where long duration therapy is needed. Longer duration echinocandin therapy with weekly dosing (e.g. Rezafungin) and novel oral formulation glucan synthase inhibitor antifungal therapy with minimal cyp interaction (e.g. Ibrexafungerp) are, however, currently in phase 3 clinical trials in an invasive fungal infection setting [72, 73]. Whether these drugs would be effective in CF fungal disease and present an alternative attractive therapeutic option within a CFTR modulator setting in the future remains to be seen.

## Conclusion

In conclusion, fungal disease in individuals with CF provides an evolving clinical challenge with new resistance and therapies developing. The advent of CFTR modulators represents a significant revolution in CF care and is likely to alter the phenotype of CF lung disease. The effect these drugs will have on the prevalence and presentation of fungal disease, and the impact of antifungal therapy in patients on CFTR modulators is not yet clear. Further research is required to systematically assess CF fungal disease and antifungal therapy as increasing numbers of patients gain access to these life-changing treatments.
